# Facing the Growing COVID-19 Infodemic: Digital Health Literacy and Information-Seeking Behaviour of University Students in Slovenia

**DOI:** 10.3390/ijerph18168507

**Published:** 2021-08-12

**Authors:** Mitja Vrdelja, Sanja Vrbovšek, Vito Klopčič, Kevin Dadaczynski, Orkan Okan

**Affiliations:** 1 National Institute of Public Health, Trubarjeva 2, 1000 Ljubljana, Slovenia; sanja.vrbovsek@nijz.si (S.V.); vito.klopcic@nijz.si (V.K.); 2 Department of Nursing and Health Science, Fulda University of Applied Sciences, 36037 Fulda, Germany; kevin.dadaczynski@pg.hs-fulda.de; 3 Centre for Applied Health Science, Leuphana University Lueneburg, 21335 Lüneburg, Germany; 4 Interdisciplinary Centre for Health Literacy Research, Faculty of Educational Science, Bielefeld University, Universitätsstraße 25, 33615 Bielefeld, Germany; orkan.okan@uni-bielefeld.de

**Keywords:** health literacy, digital health literacy, information-seeking, communication digital behaviours, students, COVID-19, infodemic, Slovenia

## Abstract

The entire world is faced with the COVID-19 pandemic, which is also accompanied by an infodemic. This refers to the rapid spread of (accurate and false) information, mainly through internet usage increasing. Digital health literacy (DHL) is therefore important for addressing challenges related to online health information and services, as well as for navigation through the complex information landscape with huge amounts of different (and conflicting) information about COVID-19. The aim of this study is to examine the level of DHL in relation to COVID-19 in Slovenian university students and to determine online information-seeking behaviour in order to plan and prepare effective communication interventions for this sub-population. A cross-sectional survey, administered by an online questionnaire, was conducted to collect data on DHL. A total of 3621 students participated, of whom 70% were female and the average age was 22.65 years (SD = 4.65). Bivariate analyses were performed to assess the association of key characteristics with DHL. Overall, the results show that the level of DHL among students is sufficient. Most difficulties were reported in assessing the reliability of information (*n* = 1484, 49.3%). Approximately one third of the students (*n* = 847, 27.9%) reported having problems in finding information of their interest, and somewhat more (*n* = 900, 29.6%) reported difficulties in making a selection among all the information found. Students with a sufficient level of DHL are more likely to seek information through search engines and websites of official institutions, while students with a limited level of DHL more frequently reported using social media for health information searches. It is necessary to establish interventions for a systematic lift of the DHL and health literacy (HL) of all population groups.

## 1. Introduction

The COVID-19 pandemic caused by the SARS-CoV-2 virus has profoundly impacted the whole world and has also significantly changed it [1]. Millions of people have been infected with the virus worldwide, many have been hospitalized due to the disease, and millions of people have died [2]. The consequences of the COVID-19 pandemic will be large and extensive throughout all areas [3].

At the beginning of the pandemic, the World Health Organization (WHO) emphasized that along with the epidemic of an infectious disease comes an infodemic [4]. During the crisis, the information landscape has become (even more) complicated, and people are more often confronted with conflicting information resulting in a high level of uncertainty through complex information environments [5]. This may lead to people acting carelessly and jeopardizing the efforts of governments and health authorities to manage COVID-19 [6] in a way that allows a sufficient level of vaccination to achieve collective immunity [7]. It can also undermine the credibility of scientific expertise with potentially longer-term consequences [8] and can strengthen the growing vaccine hesitancy [9,10,11,12], which is very alarming because it can have a significant impact on the effectiveness of communicable disease management in the future.

Due to the SARS-CoV-2 virus pandemic, the need for information has increased, so that people are searching for information even more frequently, and at the same time (co)creating it themselves and making it available to others via online social networks. All this leads to an abundance of information and has helped strengthen the infodemic, which is not a new phenomenon [13,14]. Infodemic refers to the rapid spread of accurate and false information (disinformation, misinformation, fake news and conspiracy theories, which are forms of false information) through social media and other outlets [15,16,17]. The term of infodemiology was first mentioned by Eysenbach in 2002 and was presented as the epidemiology of (mis)information. Information epidemiology or infodemiology identifies areas where there is a knowledge translation gap between best evidence (what some experts know) and practice (what most people do or believe), as well as markers for “high-quality” information [18]. The term is now widely used by the World Health Organization [19], which is aware of consequences of the infodemic. It can lead to distress by information, an increased tendency to avoid information and avoiding of preventive measures. Thus, distress may generally induce adaptive behaviour in support of crisis management, unless individuals respond to it by avoiding information [20].

During this current pandemic, people have found themselves overwhelmed with news containing (fake) reports and (mis/dis)information, and they had neither the time nor the competencies to understand it correctly [21]. They tend to navigate the complex information environments marked by high levels of uncertainty [5] in order to remain healthy and take relevant precautions using the information available [6]. Therefore, dealing with complex health information requires adequate health literacy (HL) [22]. It can help fight the pandemic, as health literate individuals tend to show a higher compliance with government regulation and recommendations with protective behaviours [23].

HL is defined as an individual’s knowledge, motivation and competencies to access, understand, appraise, and apply health information, as well as make decisions regarding health information, especially in relation to health promotion, prevention and health care at all stages of life [24,25,26]. Therefore, HL is crucial at both the individual, as well as the societal level [15]; namely, increasing HL is an important intervention for improving the state of health of the entire population [25].

HL is an important determinant of health. It enhances people’s knowledge, adapts their existing healthy lifestyles, reduces carelessness, prevents over-reactions (such as panic reactions), and adopts preventive behaviours [27,28,29]. It can be seen as a major resource in dealing with health information, which is even more important in the COVID-19 pandemic. This is confirmed by a representative German study, which showed that 50% of the adult population ≥16 years have difficulties in dealing with COVID-19-related health information in their everyday lives [30]. Low levels of HL are associated with poorer levels of knowledge and a poor self-care capacity in patients, poorer use of health services, poorer health and shorter life expectancy [31], and also with increased healthcare costs [32]. Strengthening HL means that individuals will be more empowered and knowledgeable about medical topics, as well as more actively involved in health decision-making, which increases the probability of better health outcomes [33]. In addition, as shown by a recent Australian study, those with inadequate HL had poorer knowledge and understanding of COVID-19 symptoms, were less able to identify an infection and prevent an infection, and experienced more difficulties in finding information and understanding messaging about COVID-19 compared to individuals with sufficient HL [34].

There is also the growing importance of digital health literacy (DHL). It refers to the ability to seek, find, understand and appraise health information from electronic sources and apply the knowledge gained to address or solve a health problem [35]. According to Sørensen, DHL is essential for saving time, costs and lives [36]. Previous research has demonstrated that high levels of self-reported DHL are associated with better health and quality of health care; more positive health behaviours, including prevention and management of chronic diseases; and increased procedural health knowledge [37]. People with limited or insufficient DHL usually find it more difficult to comprehend health information [38]. Recent studies from Germany and Portugal demonstrated that even those people with satisfactory level of HL and DHL have difficulties in judging whether they can trust health information found online and on social media [30,39]. There is a significant positive association between having sufficient levels of some subdimensions of DHL and the sources used for information search (e.g., website of public bodies) [40].

Therefore, the development of HL is essential for empowering individuals to understand and use online information in an effective and health-promoting way [41]. As demonstrated, DHL directly and indirectly affects health. The direct relationship concerns DHL and health behaviours, such as healthy eating, exercise, and sleep [42,43]. The indirect paths lead from DHL to information-seeking behaviour which has an effect on health (social media use is negatively associated with mental health) [44]. Due to the digital transformation in late modern society, DHL is becoming increasingly relevant for people to take health-related decisions [45], especially in the time of the COVID-19 pandemic when numerous activities and services moved to the Internet. It is important to emphasize that people with higher DHL are less likely to be infected with COVID-19, and higher DHL is also associated with higher well-being and self-efficacy for pandemic-related stress [38]. At the same time, new technologies are constantly emerging that can make a significant contribution to strengthening DHL skills and improve health outcomes [46]. Today’s young people are people born after the year 2000 [47], who represent a generation that has grown up with new technology and have spent their lives surrounded by many digital tools that are an integral part of their lives [48]. As a result of this, today’s students think and process information differently from their predecessors [49]. Many young adults use the internet as their primary source of health information, spend a large amount of time online, utilize more and different social media networks, and show trust in digital information [50]. A recent survey among Slovenian university students showed that they use the Internet for an average of 286 min a day during the week, which is slightly less than five hours a day, and on average 282 min a day during the weekend, which is only slightly less than on weekdays [51]. This is another reason why young people have better access to digital information compared to older population groups [52], so based on their experience, it is not surprising that young people have higher DHL than other population groups [23]. More recent international studies showed that DHL among university students is well-developed, but there is still a significant proportion of students facing difficulties with some dimensions of HL [39,40,53]. Next to age, there are also other sociodemographic factors that are associated with students’ DHL, such as socioeconomic status (SES) and gender. While there a clear tendency towards a higher frequency of limited DHL for individuals with lower SES [54], the evidence related to gender differences of HL remain mixed, with some studies indicating a higher HL for women [55,56,57,58].

The aim of this article was to examine the level of COVID-19 related DHL in the Slovenian university student population. Further, we wanted to ascertain the level of DHL in relation to socio-demographic factors and to determine which online sources they use and how often, what is relevant regarding information, and how satisfied they are with the information they find. In addition, the study examined the differences between DHL and the use of different online sources, the perceived importance of different aspects of the information obtained and the satisfaction with the information found.

## 2. Materials and Methods

### 2.1. Study Design and Study Population

**Figure 1:**
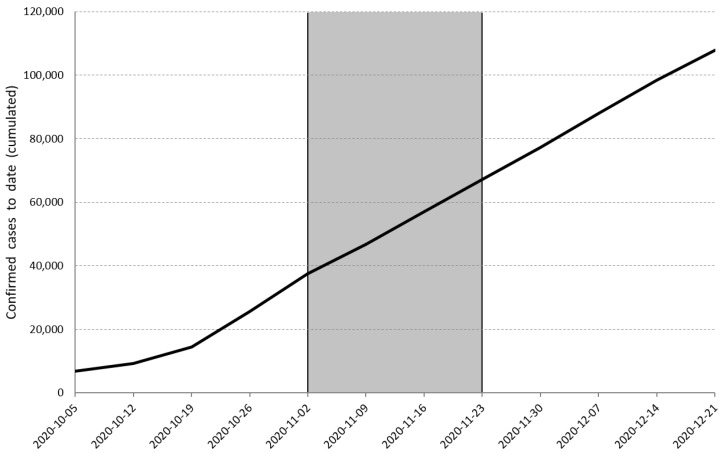
Confirmed cumulative cases of COVID-19 in Slovenia from March 2020 to May 2021. Note: Grey area marks the timing and duration of the survey.

The study is a part of the COVID-HL Consortium, a network of more than 50 countries, which conducted a university students survey based on the same questionnaire, which will undergo international comparisons [59]. As far as we know, this is the first study of DHL among the university student population in Slovenia. This cross-sectional survey was conducted among Slovenian university students from 2 to 23 November 2020, when incidence and mortality rates increased substantially in Slovenia. During that time, the total number of confirmed cases of COVID-19 infections in Slovenia increased from 37,396 (2 November 2020) to 67,106 (23 November 2020), which means that the number of confirmed cases almost doubled during this time alone. The increase in the number of cases is seen in Figure 1. According to the National Institute of Public Health, 765 people died of COVID-19 in Slovenia during this period and by the beginning of July 2021, a total of more than 4700 inhabitants of Slovenia had died, which is quite a lot considering the fact that Slovenia has 2 million inhabitants.

The questionnaire was delivered in the form of an online survey, designed with the 1KA or EnKlikAnketa (https://www.1ka.si/ accessed on 24 October 2020) online survey tool. A link to the survey was included in the invitation to participate. Before completing the survey, the respondents were informed about the aim of the survey, that their participation was voluntary and could be revoked without justification at any point, and that their confidentiality and anonymity were fully protected in the survey.

A non-probability sample was realized including a two-step invitation procedure; the first step included invitations to participate that were sent via e-mail to all faculties and colleges in Slovenia, including 16 colleges, 75 faculties, 9 independent higher education institutions and 2 postgraduate schools across Slovenia. All universities were asked to forward the invitation to their students by using internal communication channels (second step). The fact that the respondent was enrolled in one of the faculties or higher education institutions served as a criterion for inclusion in the sample for this study. Those who completed the survey but were not students were excluded.

After data cleaning and consistency checking, the final sample included 3621 respondents aged between 18 and 63 years, with an average age of 22.65 years (SD = 4.65). Based on a basic population of 71,957 university students in Slovenia (30 October 2020), this corresponds to 5%. Of these, 70.0% were women and 30.0% were men. Compared to the general population, male university students were slightly underrepresented in our sample (30% vs. 39.5%). The distribution of respondents by sociodemographic characteristics can be seen in Table 1.

More than half of the respondents were at the first (Bologna) level of study (56.1%) and almost two thirds reported a middle subjective social status (64.7%).

### 2.2. Measures

To allow cross-country comparisons, the same COVID-HL survey instrument was used across all participating countries. Necessary translations were made in accordance with standardized procedures suggested by WHO [60].

For the purposes of this article, we collected data on the sociodemographic characteristics of the respondents (gender, age, current level of studies, and subjective social status (SSS)). Respondents were divided into four categories according to their age: (1) 20 years or less; (2) 21–23 years; (3) 24–26 years; and (4) 27 years or more.

In terms of study level, we divided students in three categories: (1) undergraduate study programme or first cycle Bologna degree; (2) master’s programme or second cycle Bologna degree; and (3) doctoral study programme or third cycle Bologna degree.

Regarding subjective social status (SSS), students indicated their overall socioeconomic position through the translated MacArthur Scale [61] from 1 (lowest status) to 10 (highest status), where they would rank compared to other inhabitants of Slovenia. Based on the answers, three categories of SSS were defined: low SSS (1 to 4), middle SSS (5 to 7), and high SSS (8 to 10) [40].

COVID-19-related DHL was determined using three subscales of the DHLI questionnaire (Digital Health Literacy Instrument, [37]) which were adapted for COVID-19 and translated from English. We used the following subscales of the DHLI: (1) searching for COVID-19-related information online; (2) assessing the reliability of COVID-19-related information; and (3) determining the personal relevance of COVID-19-related information. Each subscale included three items, which could be answered on a scale from 1 (”Very difficult”) to 4 (”Very easy”). The reliability of these three subscales, calculated using Cronbach’s alpha, ranged between 0.77 and 0.79.

To assess the frequency of use of online sources when searching for COVID-19-related information, a question including ten sources was used (e.g., search engines, websites of official institutions, etc.). Frequency of use could be rated on a four-point scale from 1 (“Never”) to 4 (“Often”), and the response option 0 (“I don’t know”) was added, which was combined with the answer “Never” in the analysis. To determine the importance of certain aspects (e.g., up-to-dateness, verification) of the information sought, a six-item question was used [62]. Response options ranged from 1 (“Not important at all”) to 4 (“Very important”). One exemplary item is “How important is it for you that … the information is verified?”

A single item captured how satisfied the respondents were with the information about COVID-19 that they found online on a scale from 1 (“Very dissatisfied”) to 5 (“Very satisfied”).

### 2.3. Statistical Analyses

In addition to univariate analyses (frequencies and descriptive statistics), we also performed bivariate analyses. To allow bivariate analysis, we dichotomized the DHL subscales based on a median split; those below or exactly at the median were classified into the group with ‘limited’ DHL, and those above the median into the group ‘sufficient’ DHL. In a final analytical step, DHL was stratified by frequency of the use of online sources, perceived importance of individual aspects of searched information, and satisfaction with the information found.

Using the chi-squared test, we then determined whether there are any differences between socio-demographic characteristics (gender, age, level of studies and SSS) and the two levels of all three DHLI subscales. Using *t*-tests, we also determined whether there are statistically significant differences between ‘sufficient’ and ‘limited’ DHLI groups in the frequency of use of online sources used for information search, and the perceived importance of individual aspects of the information sought or in satisfaction with the information found. 

All analyses with *p* < 0.5 were considered statistically significant. However, since we had a relatively large sample, a particular association may prove to be statistically significant even though it has a negligible effect. Therefore, we also used Cramér’s V (for the chi-squared test) or Cohen’s d (for *t* tests) to calculate the size of the effect. For Cramér’s V, the effect is small at ≥0.1, medium at ≥0.3, and large at ≥0.5 [63]. For Cohen’s d, the following interpretation was used: ≥0.2 (small), ≥0.5 (medium), and ≥0.8 (large) [63]. For values below the threshold for small effects, we conclude that the difference (although significant) is practically negligible.

## 3. Results

Table 2 shows the frequencies of all items on DHL in connection with COVID-19. In summary, most difficulties can be found for the ability to assess the reliability of the information found, while the least difficulties are reported in the area of determining the personal relevance of health information.

Respondents reporting difficulties (answered either “Very difficult” or “Difficult”) in the dimension of “information search” range from 9.5% to 29.6%. The percentage of respondents reporting problems in the subscale of evaluating reliability ranges from 19.1% to 49.3%, while between 13.6% and 17.7% report difficulties in determining the personal relevance of the information retrieved.

The data showed that the DHL subdimension evaluation reliability is the most problematic for students, as the mean value for this dimension is 2.77 (SD 0.67), whereas the mean value for the subdimension information search is 3.0 (SD 0.56), and for the subdimension determining relevance it is 3.08 (SD 0.56), which makes it the highest across all subdimensions.

Table 3 shows the distribution of limited versus sufficient DHL stratified for socio-demographic characteristics (gender, age, level of study, SSS) and the statistical characteristics of the relationship between each sociodemographic variable, and classification in a particular DHL group calculated using the chi-squared test.

Those with higher SSS reported significantly less difficulties on all three DHLI subscales. For the other three socio-demographic variables, significant differences occurred only on the subscales of information search and evaluating reliability. Females reported more difficulties, while older respondents and those with a more proficient study level reported less difficulties on these two subscales.

With most of the statistically significant differences, Cramér’s V is less than 0.1, which means that they are trivial.

There were statistically significant and statistically relevant (as they reach the small effect size threshold) differences in gender, age, and level of studies on the subscale of assessing the reliability of information, with females reporting a slightly lower DHL (*χ*^2^ = 52.42, *p* < 0.001, *V* = 0.13). However, with increasing age (*χ*^2^ = 43.91, *p* < 0.001, *V* = 0.12) or level of study (*χ*^2^ = 33.42, *p* < 0.001, *V* = 0.11), less difficulties in DHL on assessing the reliability of information are reported in our sample.

The difference in gender (*χ*^2^ = 28.42, *p* < 0.001, *V* = 0.10) was also statistically significant, and relevant in the information search subscale were females again reporting a slightly lower DHL.

**Figure 2:**
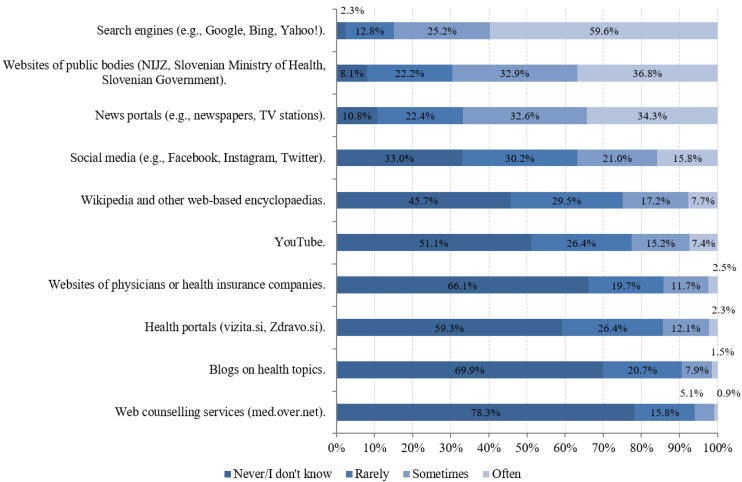
Frequency of use of internet sources for COVID-19 information-seeking (*n* = 3041 to 3053).

**Figure 3:**
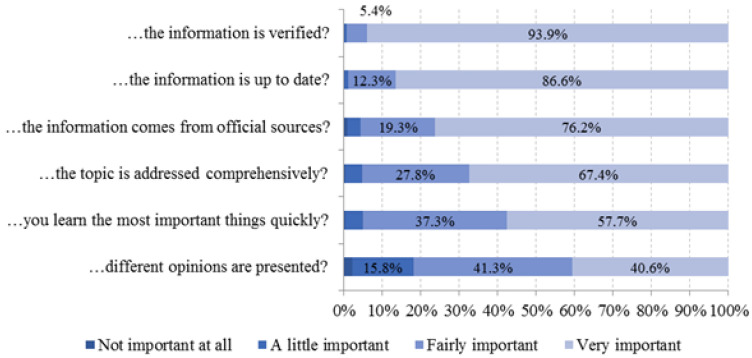
Frequency of perceived importance of aspects in COVID-19 information-seeking. ‘How important is it for you that …’ (*n* = 3045 to 3052).

Figure 2 and Figure 3 show the proportions of the respondents’ answers to questions regarding the frequency of use of online sources and the importance of individual aspects of the information sought.

As shown in Figure 2, respondents most often used search engines to search for COVID-19-related information (84.8%), followed by websites of public bodies, such as the National Institute of Public Health (NIJZ), the Ministry of Health of the Republic of Slovenia, the Government of the Republic of Slovenia (69.7%), and media portals (66.9%). In contrast, online consultation (6%), blogs on health topics (9.4%), and websites of doctors or health insurance companies (14.2%) were least frequently used.

As depicted in Figure 3, almost all respondents (99.3%) perceived it as fairly and very important that the information is verified. In contrast, 18% of respondents attach no or only little importance to the issue that different opinions are presented in the information obtained.

Regarding the satisfaction with the information found on COVID-19 online, 4.1% reported that they were very dissatisfied and 9.0% were dissatisfied. Furthermore, almost half of the respondents (49.3%) reported that they were only partially satisfied with the information they find on COVID-19 online, 35.0% were satisfied and 2.6% were very satisfied with the information they find.

Table 4 shows that respondents with sufficient DHL regarding information search used search engines, websites of public bodies and Wikipedia significantly more frequently than respondents with limited DHL. In turn, those having a lower DHL used social media, blogs, web counselling services and health portals significantly more frequently. Respondents with sufficient DHL on this subscale deemed up-to-date, verified and comprehensively addressed information significantly more important than respondents with limited DHL, while the latter attached a higher importance to the fact that different opinions are presented.

Respondents with sufficient DHL on the subscale of evaluating the reliability of health information used websites of public bodies and Wikipedia significantly more frequently, and deemed up-to-date information as significantly more important, than respondents with limited DHL on this subscale. The latter used social media, blogs, web counselling services and health portals significantly more frequently and deemed it significantly more important that different opinions are presented.

Table 5 shows a similar pattern in the frequency of use of online sources, with respondents with limited DHL, regarding determination of the relevance of information, using social media, blogs, web counselling services and health portals significantly more frequently than respondents with sufficient DHL on this subscale. The latter used only websites of public bodies significantly more frequently, but they deemed up-to-date, verified, comprehensively addressed information and information that comes from official sources as significantly more important than respondents with limited DHL. In turn, university students with limited ability to determine personal relevance perceived the presentation of different opinions as significantly more important.

Both Table 4 and Table 5 show that respondents with sufficient DHL were significantly more satisfied with the information they find than their counterparts with limited DHL.

Even though the difference between the groups in frequency of the use of online sources, perceived importance of individual aspects of information searched, and satisfaction with the information found is statistically significant, the threshold for the small effect size (Cohen’s d) is reached only in seven comparisons and consequently only these seven differences are considered as relevant.

Those with sufficient DHL on the subscale of information reliability used websites of public bodies (*t* = 6.51, *p* < 0.001, *d* = 0.24) and Wikipedia or other online encyclopedias (*t* =6.26, *p* < 0.001, *d* = 0.23) as a search resource significantly more often, and used social media (*t* = −6.89, *p* < 0.001, *d* = −0.25) significantly less often as a source of information than respondents with limited DHL. Those with sufficient DHL on the subscale for determining the adequacy of information also used the websites of public bodies (*t* = 6.41, *p* < 0.001, *d* = 0.24) more often than respondents with limited DHL. 

Small effect sizes were also found for information satisfaction on all three DHL subscales, information search (*t* = 12.58, *p* < 0.001, *d* = 0.48), evaluating information reliability (*t* = 12.46, *p* < 0.001, *d* = 0.46) and determining relevance of information (*t* = 10.23, *p* < 0.001, *d* = 0.39). On the subscale of information search, the Cohen’s d coefficient narrowly misses the threshold for a medium effect size. In all three subscales, those in the sufficient DHL group are more satisfied with the information they find. 

## 4. Discussion

The cross-sectional study contributes to the literature on DHL in general, and provides valuable insights into COVID-19-related DHL among Slovenian university students and their online information-seeking behaviour. The study findings are also important with regard to possible interventions designed to prevent and curb the spread of the coronavirus. In general, the results indicate that DHL levels among students are sufficient. Of all the DHL questions used, the percentage of respondents reporting no difficulties fell below 70% on only two questions (both within the reliability assessment subscale), which is similar to some other studies [12,41,51]. Given that higher DHL is associated with higher well-being and self-efficacy for pandemic-related stress [53], this finding is promising. At the same time, people with higher DHL are less likely to be infected with COVID-19 [53], while other studies also show that students are clearly very confident that they are able and skilled enough to navigate through the digital health information landscape [23]. 

The Internet provides constant access to all types of (accurate and false) information from various sources, with individuals having, to some extent, autonomous, independent, anonymous, and free access to health information. Although the information is very easily accessible, the question arises as to how to find high quality information and how to navigate through the information complexity. It is an interesting paradox. Our research results showed that about a third of the surveyed Slovenian students (847 students or 27.9%) have difficulties in finding the information they are interested in, and a slightly higher percentage (900 students or 29.6%) find it difficult to choose from all the information they find. This points to the fact that there is a great deal of information about the coronavirus and that even young people have difficulties assessing it, although they are very experienced at using digital media [64,65]. This is also confirmed by the finding that as many as half of our respondents (1484 students or 49.3%) have difficulties assessing the reliability of information, which is seven percent more than found in the German COVID-HL survey (42.3%) [37], which means that this is the most difficult task. Concerning the use of the information obtained, a high level of DHL is shown; 85.4% of respondents reported not having problems assessing whether the information is useful to them, 82.4% reported having no problems in using information in everyday life, and 86.4% reported using it to make decisions about their own health. Based on these findings, we consider that there is a need for the establishment of a national website managed by a professional institution (these usually have a higher level of trust than governmental institutions), which would be a uniform online information platform. In order to avoid a lengthy process of validating and reviewing the various issues that need to be communicated in a timely manner in a crisis situation such as COVID-19 pandemic, it would make sense to form a cross-sectional group of experts. It would prepare various information to communicate on a uniform website and also on social media, where a strategic communication approach is needed, based on timely proactive communication and rapid reactive communication in order to address the issue of dis-/misinformation that often arises on social media. WHO, other EU organizations and governments established information portals, dedicated to the dissemination of COVID-19 information to increase HL among different population groups (such as covid19.who.int, cdc.gov, ecdc.europa.eu, www.government.nl/topics/coronavirus-covid-19, www.santepubliquefrance.fr/dossiers/coronavirus-covid-19 etc all accessed on 24 July 2021). These could mitigate some of the DHL-related problems in different population groups, both among students and also the general population. In Slovenia, a national website for COVID-19 vaccination was established (www.cepimose.se accessed on 24 July 2021) and is edited by the National Institute of Public Health. The website has (with intensive communication) become very known and is being frequently visited (according to Google analytics, from 1st of February to 26th of July, the website had 1.08 million views and more than 253,000 users). 

Therefore, communication could be the key. The authorities should provide information in a comprehensible way, recognizing that people and groups with low HL may need more explanation and different communication formats, such as animations, to explain the virus, the disease, its transmission and protective measures [65]. There is also a need for increased public health communication and online engagement through existing channels [66]. Targeted communication is also important for better navigation of information environments during the infodemic, identification of mis/dis-information, and decision-making based on reliable and trustworthy information [30]. In any case, an open and transparent communication can be crucial in all of this; namely, it can strengthen trust in official information sources, because without trust it is not possible to communicate effectively and efficiently.

Regarding the sources of information about the novel coronavirus, we found that students with sufficient DHL (specifically with regard to the reliability subscale) use websites of official institutions and search engines (e.g., Google) more frequently, while students with limited abilities to critically assess online health information are more likely to search for information through social media [67]. Studying how people behave online, how and what information they search for, how they navigate the web and the mess of COVID-19-related (mis/dis)information are valuable insights into an individual’s health-related behaviour [13]. It is crucial to know the individuals’ communication behaviours [68], especially among the young adults who often use digital platforms. Therefore, critical HL has never been more important than in these times of pandemic, when we are witnessing a large surplus of information and high expectations regarding (self-)control over health [29]. This particularly points toward the need to address this dimension of HL in a more focused manner and earlier on in schools by, e.g., training pupils in how to critically analyze information and sources, critically read health messages, news and claims, etc.

Our research further finds that the level of information satisfaction is significantly higher in university students with sufficient DHL. The results of our study are similar to the COVID-HL survey in Pakistan, where the majority of students were satisfied or partly satisfied with information regarding the coronavirus [53]. There are probably a number of reasons for this. One reason could be that those with higher DHL are more able to search, understand, assess and use the information they are looking for, and that they are also more persistent in finding correct and reliable information and are not satisfied with just the first information they encounter (and not evaluating the source).The reasons can also be related to the perceived benefits, the costs and the user (past) experience [69]. This also raises calls for further research to understand predictors not only of information search, but also of information satisfaction. 

In addition, given that university students are among the more educated and more skilled users of digital platforms, the question arises about the DHL levels of other population groups that are less educated and more vulnerable. Given the importance (digital) HL has for preventive behaviour and also for vaccination-related attitudes and behaviours, interventions that aim to improve HL could make an important contribution to overcome the pandemic as soon as possible.

To our knowledge, this is the first study investigating DHL and aspects of online information-seeking behaviour of university students in Slovenia. However, our study also has certain limitations. The main limitation of the study is that no statistical analysis was performed to find out any correlation between DHL and the ability to find correct and high-quality information and trust in information sources. Because of this, we cannot be certain if the subscales of DHL adequately represent an individual’s actual digital HL or just their subjective assessment of it. Second, in our study we focused mainly on the sources used for information search, and further empirical research should take into account more dimensions of information-seeking behavior such as attitudes, social norms or usability [70,71]. Third, we did not have probability sampling, nor did we weight the sample, which means that the sample is not representative of the student population in Slovenia. Since the study was conducted at the beginning of the new academic year and lasted only three weeks, it is quite possible that it did not include those most affected by the pandemic and who may have even had to stop their studies in the previous academic year. We also relied on the management of university faculties to provide the students with the questionnaire. Universities differed in their approach to this inquiry, so there may have been differences in reach here as well. As the questionnaire was only available in an online format, we may have excluded those with lower access to digital devices or digital literacy and are probably also the members of the most vulnerable groups. Another limitation may be that, according to their results on individual subscales, we divided individuals into two groups (‘limited’ and ‘sufficient’) by means of median split. This procedure is based solely on an artificial division of the sample and results in loss of information, including the risk that individuals with similar mean values are divided into two different categories. 

## 5. Conclusions

The study represents an important implication for planning communication interventions for the student population in Slovenia—from the current COVID-19 perspective, as well as for other public health activities in the future. The study shows that a proportion of Slovenian university students have difficulties in finding and making the selection of health information. They also have problems with assessing the reliability of information. Therefore, there is a specific need for interventions that promote the skills that allow critical finding and evaluation of health information retrieved online. In this context, the specific needs of female and younger university students, as well as those with lower SSS, should be taken into account. We suggest that facing the infodemic should become an important public health priority in the future, because it will not cease to exist with the end of COVID-19 pandemic. Additionally, public health activities should not only focus on the individual abilities, but interventions should also focus on information providers and technical solutions to identify misleading health information. From the perspective of the first “infodemiologist”, there are four pillars of infodemic management: (1) information monitoring; (2) building eHealth literacy and science literacy capacity; (3) encouraging knowledge refinement and quality improvement processes, such as fact-checking and peer-review; and (4) accurate and timely knowledge translation, minimizing distorting factors such as political or commercial influences [72].

## Figures and Tables

**Table 1:** Socio-demographic characteristics of the study sample.

	Total *n* (%)	Women *n* (%)	Men *n* (%)
Age (*n* = 3621)			
20 years or less	1233 (34.1)	870 (34.3)	363 (33.4)
21–23 years	1414 (39.0)	983 (38.8)	431 (39.7)
24–26 years	657 (18.1)	469 (18.5)	188 (17.3)
27 years or more	317 (8.8)	213 (8.4)	104 (9.6)
Level of study (*n* = 3620)			
1st Bologna	2032 (56.1)	1388 (54.8)	644 (59.3)
2nd Bologna	1519 (42.0)	1109 (43.7)	410 (37.8)
3rd Bologna	69 (1.9)	37 (1.5)	32 (2.9)
SES (*n* = 3621)			
Low	518 (14.3)	384 (15.1)	134 (12.3)
bottom	2344 (64.7)	1663 (65.6)	681 (62.7)
High	759 (21.0)	488 (19.3)	271 (25.0)
Total	3621 (100)	2535 (70.0)	1086 (30.0)

**Table 2:** Frequencies for all DHLI items.

Items	Very Difficult	Difficult	Easy	Very Easy
Information search				
make a choice from all the information you find? (*n* = 3034)	3.4%	26.2%	52.6%	17.8%
use the proper words/search query to find the information you are looking for? (*n* = 3040)	1.0%	8.5%	53.2%	37.3%
find the exact information you are looking for? (*n* = 3041)	4.5%	23.3%	51.9%	20.2%
Evaluating reliability				
decide whether the information is reliable or not? (*n* = 3009)	10.3%	39.0%	40.9%	9.8%
decide whether the information is written with commercial interests? (*n* = 3007)	7.3%	30.0%	40.7%	22.0%
check different websites to see whether they provide the same information? (*n* = 2996)	2.5%	16.6%	55.6%	25.3%
Determining relevance				
decide if the information you found is applicable to you? (*n* = 3026)	1.4%	13.2%	61.3%	24.2%
apply the information you found in your daily life? (*n* = 3019)	2.2%	15.5%	61.2%	21.1%
use the information you found to make decisions about your health? (*n* = 3016)	2.0%	11.6%	55.9%	30.5%

**Table 3:** DHL stratified by sociodemographic characteristics.

	Information Seeking					Evaluating Reliability					Determining Relevance				
Characteristic	Limited *n* (%)	Sufficient *n* (%)	*χ*^2^ (df)	*p*	*V*	Limited *n* (%)	Sufficient *n* (%)	*χ*^2^ (df)	*p*	*V*	Limited *n* (%)	Sufficient *n* (%)	*χ*^2^ (df)	*p*	*V*
Gender			28.42 (1)	<0.001	**0.10**			52.42 (1)	<0.001	**0.13**			3.67 (1)	0.056	NA
Female	1445 (67.9)	684 (32.1)				1169 (55.8)	927 (44.2)				1368 (64.8)	743 (35.2)			
Male	521 (57.8)	381 (42.2)				368 (41.3)	523 (58.7)				544 (61.1)	346 (38.9)			
Age			18.61 (3)	<0.001	0.08			43.91 (3)	<0.001	**0.12**			3.49 (3)	0.323	NA
20 years or less	688 (69.1)	307 (30.9)				561 (57.5)	415 (42.5)				646 (65.5)	340 (34.5)			
21–23 years	768 (64.4)	424 (35.6)				610 (51.9)	566 (48.1)				748 (63.4)	432 (36.6)			
24–26 years	362 (62.4)	218 (37.6)				273 (47.6)	301 (52.4)				362 (63.2)	211 (36.8)			
27 years or more	148 (56.1)	116 (43.9)				93 (35.6)	168 (64.4)				156 (59.5)	106 (40.5)			
Level of study			7.88 (2)	0.019	0.05			33.42 (2)	<0.001	**0.11**			1.25 (2)	0.536	NA
1st Bologna	1056 (65.7)	552 (34.3)				886 (56.3)	687 (43.7)				1022 (64.5)	562 (35.5)			
2nd Bologna	879 (64.6)	481 (35.4)				628 (46.5)	723 (53.5)				852 (62.9)	502 (37.1)			
3rd Bologna	30 (48.4)	32 (51.6)				23 (37.1)	39 (62.9)				37 (59.7)	25 (40.3)			
SSS			10.28 (2)	0.006	0.06			11.25 (2)	0.004	0.06			10.05 (2)	0.007	0.06
Low	288 (67.9)	136 (32.1)				217 (52.5)	196 (47.5)				279 (66.7)	139 (33.3)			
Middle	1310 (65.9)	679 (34.1)				1042 (53.1)	920 (46.9)				1272 (64.7)	693 (35.3)			
High	368 (59.5)	250 (40.5)				278 (45.4)	334 (54.6)				361 (58.4)	257 (41.6)			

Note: Relevant effect sizes are highlighted in bold. *χ*^2^ is chi-square, df are degrees of freedom, *p* is *p*-value, and *V* is Cramér’s V.

**Table 4:** DHL regarding the frequency of use, perceived importance, and information satisfaction.

	Information Seeking				Evaluation Information Reliability			
Item	Limited, Mean (SD)	Sufficient, Mean (SD)	*p*	*d*	Limited, Mean (SD)	Sufficient, Mean (SD)	*p*	*d*
Internet sources								
Search engines (e.g., Google, Bing, Yahoo!).	3.40 (0.81)	3.47 (0.79)	0.017	0.09	3.41 (0.80)	3.44 (0.79)	0.228	NA
Web sites of public bodies (NIJZ, Slovenian Ministry of Health, Slovenian Government).	2.95 (0.95)	3.05 (0.97)	0.008	0.10	2.88 (0.97)	3.10 (0.92)	<0.001	**0.24**
Wikipedia and other web-based encyclopaedias.	1.82 (0.92)	1.96 (1.02)	<0.001	0.15	1.77 (0.92)	1.99 (0.99)	<0.001	**0.23**
Social media (e.g., Facebook, Instagram, Twitter).	2.26 (1.06)	2.08 (1.07)	<0.001	−0.16	2.32 (1.07)	2.05 (1.04)	<0.001	**−0.25**
YouTube.	1.78 (0.95)	1.81 (0.97)	0.443	NA	1.76 (0.97)	1.82 (0.94)	0.101	NA
Blogs on health topics.	1.43 (0.71)	1.37 (0.68)	0.014	−0.09	1.46 (0.74)	1.36 (0.66)	<0.001	−0.14
Web counselling services (med.over.net).	1.31 (0.61)	1.25 (0.57)	0.016	−0.09	1.31 (0.62)	1.26 (0.58)	0.017	−0.09
Health portals (vizita.si, Zdravo.si).	1.61 (0.80)	1.51 (0.76)	0.001	−0.13	1.61 (0.81)	1.54 (0.77)	0.011	−0.09
Websites of physicians or health insurance companies.	1.50 (0.78)	1.51 (0.83)	0.734	NA	1.47 (0.76)	1.55 (0.83)	0.008	0.10
News portals (e.g., newspapers, TV stations).	2.90 (0.98)	2.92 (1.02)	0.505	NA	2.90 (0.97)	2.91 (1.02)	0.854	NA
Importance								
information is up to date	3.83 (0.42)	3.89 (0.34)	<0.001	0.14	3.83 (0.41)	3.89 (0.36)	<0.001	0.15
information is verified	3.92 (0.31)	3.95 (0.23)	0.019	0.09	3.93 (0.30)	3.94 (0.25)	0.166	NA
you learn the most important things quickly	3.53 (0.6)	3.53 (0.59)	0.989	NA	3.52 (0.61)	3.53 (0.59)	0.753	NA
the information comes from official sources	3.70 (0.58)	3.73 (0.56)	0.288	NA	3.70 (0.59)	3.73 (0.55)	0.078	NA
different opinions are presented	3.23 (0.77)	3.16 (0.80)	0.018	−0.09	3.26 (0.75)	3.14 (0.81)	<0.001	−0.15
the topic is addressed comprehensively	3.60 (0.60)	3.66 (0.57)	0.018	0.09	3.61 (0.59)	3.63 (0.58)	0.481	NA
Satisfaction	3.10 (0.76)	3.48 (0.83)	<0.001	**0.48**	3.06 (0.79)	3.42 (0.79)	<0.001	**0.46**

Note: Relevant effect sizes are highlighted in bold. NA, not applicable; SD, standard deviation; *p*, significance level; *d*, Cohen’s d coefficient.

**Table 5:** DHL regarding the frequency of use of internet sources, perceived importance of individual aspects of the information sought, and information satisfaction.

	Determining Relevance of Information			
Item	Limited, Mean (SD)	Sufficient, Mean (SD)	*p*	*d*
Internet sources				
Search engines (e.g., Google, Bing, Yahoo!).	3.41 (0.81)	3.45 (0.78)	0.147	NA
Web sites of public bodies (NIJZ, Slovenian Ministry of Health, Slovenian Government).	2.90 (0.95)	3.13 (0.95)	<0.001	**0.24**
Wikipedia and other web-based encyclopaedias.	1.85 (0.94)	1.91 (0.99)	0.110	NA
Social media (e.g., Facebook, Instagram, Twitter).	2.26 (1.06)	2.06 (1.05)	<0.001	−0.19
YouTube.	1.81 (0.97)	1.76 (0.94)	0.261	NA
Blogs on health topics.	1.45 (0.73)	1.34 (0.65)	<0.001	−0.15
Web counselling services (med.over.net).	1.31 (0.61)	1.25 (0.58)	0.007	−0.10
Health portals (vizita.si, Zdravo.si).	1.60 (0.80)	1.53 (0.78)	0.021	−0.09
Websites of physicians or health insurance companies.	1.49 (0.77)	1.54 (0.85)	0.111	NA
News portals (e.g., newspapers, TV stations).	2.90 (0.98)	2.92 (1.02)	0.533	NA
Importance				
information is up to date	3.83 (0.42)	3.90 (0.34)	<0.001	0.18
information is verified	3.93 (0.30)	3.95 (0.24)	0.021	0.09
you learn the most important things quickly	3.53 (0.60)	3.51 (0.59)	0.441	NA
the information comes from official sources	3.69 (0.58)	3.75 (0.55)	0.011	0.10
different opinions are presented	3.23 (0.76)	3.16 (0.82)	0.018	−0.09
the topic is addressed comprehensively	3.60 (0.60)	3.66 (0.58)	0.009	0.10
Satisfaction	3.12 (0.78)	3.43 (0.82)	<0.001	**0.39**

Note: Relevant effect sizes are highlighted in bold. NA, not applicable; SD, standard deviation; *p*, significance level; *d*, Cohen’s d coefficient.

## Data Availability

Data will be made available upon request.
